# Detection of Circulating Tumor Cells in Non-Small Cell Lung Cancer

**DOI:** 10.3389/fonc.2015.00207

**Published:** 2015-09-22

**Authors:** Annkathrin Hanssen, Sonja Loges, Klaus Pantel, Harriet Wikman

**Affiliations:** ^1^BMT with Section of Pneumology, Department of Tumor Biology, University Medical Center Hamburg-Eppendorf, Hamburg, Germany; ^2^BMT with Section of Pneumology, Department of Hematology and Oncology, University Medical Center Hamburg-Eppendorf, Hamburg, Germany

**Keywords:** circulating tumor cells, non-small cell lung cancer, EpCAM, liquid biopsy

## Abstract

Lung cancer is the most common cause of cancer-related deaths that frequently metastasizes prior to disease diagnosis. Circulating tumor cells (CTCs) are found in many different types of epithelial tumors and are of great clinical interest in terms of prognosis and therapy intervention. Here, we present and discuss epithelial cell adhesion molecule-dependent and -independent capture of CTCs in non-small cell lung cancer (NSCLC) and the clinical relevance of CTC detection and characterization. Taking blood samples and analyzing CTCs as “liquid biopsy” might be a far less invasive diagnostic strategy than biopsies of lung tumors or metastases. Moreover, sequential blood sampling allows to study the dynamic changes of tumor cells during therapy, in particular the development of resistant tumor cell clones.

## Introduction

Lung cancer is one of the most frequent and deadly diseases (1). Current therapeutic strategies in particular for non-small cell lung cancer (NSCLC) are chemotherapy and drugs targeting specific molecular targets and pathways (2). The number of specific targets is increasing and about a third of NSCLC especially adenocarcinomas have specific targets, such as EGFR or ALK-rearrangements. Since these therapeutic targets are only expressed in a subset of tumors or their expression is heterogeneous within the tumor, the development of companion diagnostics that can be used for stratification and/or monitoring of targeted therapies in individual patients is of utmost importance for future drug development (2). Their development, however, is hampered by limited availability of tumor tissue due to biopsy-associated risks to the patient and the limited representation of tumor heterogeneity. Furthermore, longitudinal sampling under treatment to monitor developing resistance toward the treatment is generally not an option if invasive biopsies are required.

Circulating tumor cells (CTCs) may become new biomarkers to fill this diagnostic gap. A sensitive isolation method for CTCs holds great promise for early detection of minimal residual disease (3). Furthermore, CTCs are discussed to be deployable as predictive biomarkers guiding individual cancer treatment strategies (personalized medicine) (4). Hence, sensitive isolation and profound molecular characterization of CTCs could serve as a “liquid biopsy” and help to improve individual treatment regimens for cancer patients (5). So far, several reports have shown that CTC counts are much higher in small cell lung cancer (SCLC) compared to those of NSCLC (4).

Here, we will discuss the current state of research on CTCs in NSCLC patients and point out future directions to implement “liquid biopsies” based on the molecular and functional characterization of CTCs into clinical practice.

## CTCs as Liquid Biomarker

Today, a large number of publications exist investigating the potential clinical use of CTCs as a so-called liquid biomarker. So far, strong evidence for CTCs as prognostic markers has been collected for different types of epithelial tumors, including, breast, prostate, and colorectal cancer (3). In addition, molecular characterization of CTCs could be used to identify therapeutic targets in inoperable patients or metastatic settings, when patients cannot be re-biopsied. In some cases, the CTC count may even be superior to that of conventional imaging methods for response monitoring (6). Moreover, sequential CTC enumeration can also provide clinically relevant information on the effectiveness and progression of a systemic treatment. Therefore, CTCs are used as biomarkers in several ongoing clinical trials (3, 7, 8).

Numerous (>50) different methods for CTC detection have been published, with highly variable detection rates, sensitivity, and specificity. Clearly two major technical hurdles have to be tackled when trying to establish a CTC detection system. First, the enrichment of single CTCs among millions of normal blood cells (leukocytes, erythrocytes, and platelets); and second, the detection of CTCs in the enriched cell fractions (3, 8).

Usually, CTC assays start with an enrichment step that optimally depletes most of blood cells to allow an easier detection of single CTCs or CTC clusters. The most commonly described enrichment methods can be divided into label-dependent and -independent methods. In the label-dependent positive selection methods, CTCs are enriched using protein markers that are expressed by CTCs but not by the surrounding blood cells. The cell surface protein epithelial cell adhesion molecule (EpCAM), which is frequently used as capture antigen, is bound either to magnetic devices, such as beads, or to some solid surface used as CTC baits. Among the EpCAM-based enrichment strategies, the semi-automated CellSearch^®^ system is the most commonly used technique, which is currently the only system that has been cleared by the U.S Food and Drug administration (FDA). For negative selection, CTCs are enriched by depleting leukocytes, usually using a CD45-antibody alone or together with antibodies against other antigens expressed on blood cells. Using this system, viable CTCs have been enriched and subsequently cultured, giving rise to stable CTC cell lines and/or injected into mice giving rise to tumors in mice (9–12). Several different label-free enrichment techniques have been published. These methods rely on some physical properties of the CTCs, such as size, density, shape, or rigidity (3, 8).

Even after a very efficient enrichment, usually several hundreds or thousands of normal blood cells (lymphocytes, endothelial cells, hematopoietic, and mesenchymal stem cells) still remain and, thus, a sensitive and specific detection system is needed. Optimally, a CTC marker should be expressed on all CTCs, but not on any type of cells found in the blood. Clearly no single marker exists that could be ubiquitously used even for specific tumor entities due to the great heterogeneity found between and within single tumors. For detection, usually molecular, immunocytological or functional-based methods, and assays are used. The molecular methods rely on either the detection of tumor-specific mRNA expression (detected by RT-PCR) or specific gene alterations (including e.g., sequencing, different types of mutation-specific PCRs or FISH) (3, 8).

The most commonly used CTC detection methodologies rely on the immunocytological fluorescence detection of tumor-associated protein markers using different cocktails of antibodies. These protein-based technologies are usually utilizing antibodies that detect epithelial markers such as different members of the keratin family, normally not expressed at detectable levels in hematopoietic cells. DNA and RNA-based technologies can be used in addition to the immunocytological detection of single CTCs for verification and molecular characterization of the CTCs. The rapid advances in both next-generation sequencing and whole-genome amplification of single cells now allow the detailed characterization of the genome or expressional landscape of individual cells (10–14). Functional assays such as the EPISPOT technology detects secreted proteins from CTCs (e.g., prostate-specific antigen or cytokeratin-19) that can be captured by specific antibodies attached to the bottom of culture dishes (3, 8).

In summary, CTCs represent a reliable prognostic factor in breast, colon, and prostate cancer patients and, e.g., the detection of CTCs by the FDA-approved CellSearch^®^ system is increasingly incorporated as a diagnostic tool to the clinics. Nonetheless, concerning NSCLC, the scientific community faces a greater challenge for both the detection and the interpretation of CTCs. Importantly, Wang et al. published recently a meta-analysis, where they included the CTC results from 1576 NSCLC patients reported in 20 publications using different CTC detection methods. This meta-analysis showed that the presence of CTCs is significantly associated with a decreased disease-free and overall survival of NSCLC patients, indicating that also in NSCLC CTCs could be used as a diagnostic tool in the clinics (15).

## CTC Detection by EpCAM-Based Methods in Lung Cancer Patients

Despite the highly aggressive nature of NSCLC, it is known that CTCs are less frequently observed in NSCLC patients compared to e.g., prostate, breast, and ovarian cancer patients when detected by EpCAM-based methods (16). Interestingly, in SCLC, EpCAM-based methods detect CTCs in most patients. Indeed, both the frequency of detected CTCs and the number of CTCs per SCLC patient are among the highest reported compared to other tumor entities (17).

Hou et al. performed CellSearch^®^ CTC analysis on patient cohorts consisting of both NSCLC and SCLC. At the time of first diagnosis, 85% of SCLC (*n* = 97) patients were CTC positive. The median number of CTCs among SCLC patients was 24 per patient (range 0–44.896 CTCs/7.5 ml of blood) (25). By contrast, the same group showed that only 21% of 101 advanced NSCLC patients were positive for CTCs (range 0–146) (see Table 1) (18). CTCs were mainly detected in stage IV patients (32% positivity), whereas stage IIIA patients were CTC negative. The low CTC detection rate in NSCLC patients was also described by other groups when using the CellSearch^®^ system. CTC positivity rates were recorded between 21 and 39% (19–22, 26). Beyond that, Marchetti et al. could detect CTCs in 41% of NSCLC patients harboring activating EGFR mutations (23). Devriese et al. isolated and detected CTCs using EpCAM-targeted magnetic beads followed by quantitative real-time PCR detecting expression of CK7 and CK19. In line with the CellSearch^®^ results, 30% of the advanced NSCLC patients showed positive expression for the marker CK7 and 9% were positive for CK19 (24).

**Table 1:** **CTC detection in patients with NSCLC using EpCAM-dependent assays**.

Group (reference)	Patient disease stage	Size of cohort	Positivity rate (%)
Krebs et al. (18)	III–IV	101	21
Hofman et al. (19)	I–IV	210	39
Tanaka et al. (20)	I–IV	152	31
Isobe et al. (21)	IV	24	33
Hirose et al. (22)	IV	33	36
Marchetti et al. (23)	EGFR mutation	37	41
Devriese et al. (24)	IIIB–IV	46	30

In conclusion, fewer CTCs can be detected in NSCLC patients especially in localized disease using EpCAM-based detection methods compared to other cancer types. However, between 25 and 50% of these early stage patients with resectable tumors develop metastatic relapse. Therefore, it is likely that CTCs in NSCLC are missed with current EpCAM-based technologies.

## EpCAM-Independent Detection of CTCs in Lung Cancer

Several different EpCAM-independent isolation techniques were applied for NSCLC by different groups (see Table 2). Krebs et al. used a size-based isolation technique (ISET) and reported a positive CTC status in 32 out of 40 stage III–IV patients, whereas CellSearch^®^ detected CTCs in the same cohort only in 9 out of 40 patients. Both methods add up to a CTC positivity rate of 85% (27). Another study that used the ISET device was performed by Hofman et al., detecting five times the CTC number compared to CellSearch^®^, with CTC positivity rates of 50 versus 21% (19). Furthermore, Hou et al. reported that circulating tumor microemboli (CTM), i.e., clusters of CTCs, were only detected with the ISET filtration device in NSCLC but not with CellSearch^®^, indicating a possible loss of EpCAM expression in CTC clusters in NSCLC (25). Interestingly, CTC clusters can be detected with the CellSearch^®^ system in SCLC (25). Hosokawa et al. reported another size-based isolation [miniaturized microcavity array (MCA)] of CTCs and subsequent detection by CK expression. They revealed a 77% CTC detection rate with a median CTC number of 13 CTCs/7.5 ml of blood (28). Another EpCAM-independent study was performed by Dorsey et al. (29). When using a telomerase-based assay for CTC detection, they revealed that 65% of the stage III patient cohort was CTC positive. This publication included a pilot study that was furthermore showing a relation between CTC burden and treatment success (29).

**Table 2:** **CTC detection in patients with NSCLC using EpCAM-independent assays**.

Group (reference)	Isolation device	Patient disease stage	Size of cohort	Positivity rate (%)
Hofman et al. (19)	ISET	I–IV	210	50
Krebs et al. (27)	ISET	III–IV	40	80
Pailler et al. (30)	ISET	IV	32	100
Hosokawa et al. (28)	MCA	IV	22	77
Nel et al. (31)	Negative depletion	II–IV	43	CK: 100
Dorsey et al. (29)	Telomerase-based	III	30	65

*ISET, isolation by size of epithelial tumor cell; MCA, minituarized microcavity array*.

Negative depletion approaches were used by Nel et al., and cells were subsequently stained for the epithelial markers CK and EpCAM, the mesenchymal marker N-Cadherin, and the stem cell marker CD133 in 43 NSCLC patients (31). The authors identified a variety of different CTC subpopulations and stated that the presence of mesenchymal CTCs (positive for N-Cadherin) and an increased ratio of stem cell-like (positive for CD133) to epithelial CTCs (positive for CK) is associated with poor treatment response. Similarly, when Pailler et al. analyzed ALK-rearranged CTCs, they observed a more mesenchymal phenotype with positivity for Vimentin and N-Cadherin but without CK expression (30). The analysis of the matched primary tumors revealed a prominent epithelial phenotype with a range from moderate to strong CK and E-Cadherin expression in the tumor tissue. At moderate expression of the epithelial markers, a significant mesenchymal marker expression was also observed. Upon application of ALK inhibitors, the number of ALK-positive CTCs decreased, suggesting that CTCs could serve for monitoring of ALK-targeted therapy in NSCLC (30).

These results underline the fact that NSCLC patients obviously can harbor different CTC subpopulations, including EpCAM-positive and EpCAM-negative cells. Further isolation attempts and phenotypic characterizations have to be done in order to fully understand the CTC composition and the clinical relevance of each subpopulation.

## Monitoring of Tumor-Specific Alterations in CTCs can Predict Therapy Response when Using Targeted Therapies in NSCLC

Detection and monitoring of cancer-specific rearrangements and mutations on CTCs has also been reported lately in a few studies. In 2012, Ilie et al. demonstrated that the patients’ ALK status can reliably be determined via FISH analysis of CTCs in NSCLC patients. In a patient cohort of 87 cases, they detected five tumor biopsies as ALK positive. All these patients had also ALK-positive CTCs (32). Similar results were also obtained by Pailler et al. who could identify ALK-positive CTCs in all patients with ALK-positive primary tumor. However, in average 63% of CTCs were ALK positive. They further studied the relation of ALK-rearranged CTCs and targeted therapy intervention with crizotinib. Serial analysis of five patients showed that the number of ALK-rearranged CTCs decreases during crizotinib therapy (30). The detection and clinical relevance of ROS1-rearrangement in CTCs of NSCLC patients undergoing crizotinib therapy has also been reported lately. In this study, it was shown that the amount of ROS1-rearranged CTCs increased after 2 weeks discontinuation of crizotinib therapy, which was consistent with the CT scan of the patients. Patients not responding to crizotinib did not show any change in the level of rearranged CTCs (33). In addition to these studies, Maheswaran et al. serially analyzed four EGFR-mutated primary NSCLC tumor patients for EGFR mutation in CTCs. Treatment with gefitinib caused in general a decline in the CTC counts. They analyzed different EGFR mutations including the mutation T790M, conferring an acquired resistance against gefitinib. The FUP blood sample revealed an increase in the proportion of CTCs with the T790M mutation, in line with the acquisition of drug resistance, and the presence of mutation correlated with reduced progression-free survival (34). These studies suggest that changes in the tumor genotype during treatment response can be representatively monitored in CTCs.

## Conclusion

The literature indicates that CTCs in NSCLC might have frequently lost or downregulated EpCAM expression, probably as consequence of EMT. Still, EpCAM-positive CTCs have been shown to be relevant for the clinical outcome (15, 18). However, as they are barely detectable in earlier disease stages, a reliable combination of non-EpCAM- and EpCAM-based CTC detection methods needs to be established and validated in clinical studies.

In addition to enumeration and the gain of prognostic information, the molecular characterization of CTCs with regard to molecular alterations relevant for therapy is now feasible (10, 13, 30, 35). In NSCLC, various molecular targets each comprising only a minor subset of patients have been identified. Thus, targeted therapy needs to be adapted to the molecular characteristics of the NSCLC cells in individual patients. Taking blood samples and analyzing CTCs as “liquid biopsy” (36) might be a far less invasive diagnostic strategy than biopsies of lung tumors or metastases. Moreover, sequential blood sampling allows to study the dynamic changes of tumor cells during therapy, in particular the development of resistant tumor cell clones. While information on genomic changes in tumor cells can also be obtained from circulating DNA fragments (34, 37, 38), the capture of CTCs allows a more in-depth characterization including functional *in vitro* and *in vivo* studies using cell culture and xenograft models (10–12). These studies will largely extend our knowledge about the biology of tumor cell dissemination in NSCLC with potential clinical implications for an improved treatment.

## Author Contributions

All authors participated in drafting and revising the manuscript.

## Conflict of Interest Statement

The authors declare that the research was conducted in the absence of any commercial or financial relationships that could be construed as a potential conflict of interest.
